# Mixed Delivery Mode Reminder Strategy to Tackle Low Participation in an e-Cohort: Randomized Controlled Trial Nested Within the Le French Gut e-Cohort

**DOI:** 10.2196/88577

**Published:** 2026-08-18

**Authors:** Jessica Soyer, Aline Thomas, Sébastien Fromentin, Anne-Sophie Alvarez, Chloé Connan, Nicolas Pons, Le French Gut Consortium, Najate Achamrah, Alexandre Cavezza, Patrick Veiga, Robert Benamouzig, Jean-Philippe Bertocchio

**Affiliations:** 1 Skezi, Les Papèteries – Image Factory, 1 Esplanade Augustin Aussedat, Annecy, 74960, France, 33 670333450; 2 MetaGenoPolis, INRAE, Université Paris-Saclay, Jouy-en-Josas, Île-de-France, France; 3 See Acknowledgments; 4 AgroParisTech, INRAE, Micalis Institute, Université Paris-Saclay, Jouy-en-Josas, France; 5 Assistance Publique – Hôpitaux de Paris, Paris, Île-de-France, France; 6 Thyroide – Tumeurs Endocrines, Pitié-Salpêtrière Hospital, Assistance Publique – Hôpitaux de Paris, Paris, Île-de-France, France; 7 Reference center for rare diseases related to calcium-phosphate metabolism disorders, Pitié-Salpêtrière Hospital, Assistance Publique – Hôpitaux de Paris, Paris, Île-de-France, France

**Keywords:** e-cohort, controlled trial, reminder strategy, response rate, email reminder, text message reminder, completion

## Abstract

**Background:**

Typically, e-cohorts are susceptible to low participation rates, undermining representativeness. Frequent reminders can be a cost-effective strategy to increase response rates. However, their effectiveness may vary depending on the delivery mode and message content.

**Objective:**

This study aimed to evaluate different reminder strategies (ie, emails and/or text messages with standard and/or institutional wording) and compare their effectiveness in increasing the response rate in a population-based e-cohort of healthy adults.

**Methods:**

We conducted a 4-arm parallel randomized controlled trial nested within the Le French Gut e-cohort (registration number 2021-A01439-32). In November 2024, we included participants who were enrolled online (SKEZIA platform) but were not yet active participants (ie, eligibility, consent form, and/or personal information questionnaire had not been completed). Computer-generated randomization was stratified by time since enrollment. We sent 3 reminders, 72 hours apart, following 4 experimental designs: group 1, standard emails only; group 2, text messages only; group 3, institutional emails only; and group 4, standard email, followed by a text message and an institutional email. The primary outcome was the 30-day completion rate of the personal information questionnaire. We also measured completion time (ie, time between reminder received and questionnaire fully completed), online login rate, login time, email opening, and click-through rate. Participants were unaware of the trial, and investigators were blinded to group allocation.

**Results:**

At the end of the trial, of the 20,487 eligible participants, 19,525 received at least 1 reminder (n=5225 in group 1; n=3858 in group 2, because n=1365 of the initial 5223 had no phone number and could not receive text messages; n=5222 in group 3; n=5220 in group 4). The per-protocol completion rate was 8.4%, with a higher rate in group 4 (9.4% vs 7.4%, 8.4%, and 8.3% for groups 1, 2, and 3, respectively; *P*<.001). Completion time was faster in group 2 (mean 6.3, SD 4.3 d) compared to groups 3 and 4 (mean 7.0, SD 3.5 and mean 7.2, SD 3.8 d, respectively; *P*=.003). The online login rate was higher, and the login time was faster in group 4 (login rate: 15.6% vs 12.1%, 14.3%, and 15.3% for groups 1, 2, and 3, respectively; *P*<.001; and login time: mean 7.1, SD 4.3 d vs mean 6.7, SD 4.3 d, mean 6.2, SD 4.6 d, mean 6.8, SD 3.9 d for groups 1, 2, and 3, respectively; *P*=.002). For emails, opening rates were similar (*P*=.87), but the click-through rate was higher for institutional emails (23.1% vs 19.6% for standard formatting; *P*<.001).

**Conclusions:**

For the first time in a specifically designed randomized controlled trial, we directly compare email-only, text message–only, and a combined reminder strategy in a large e-cohort. While previous studies focused on specific populations, our work examines a general adult population, demonstrating that a mixed delivery mode strategy effectively increases response rate by 27% compared to a single-channel approach. Text messages yielded faster responses, and institutional emails, with formal wording and coordinator’s signature, seemed more appealing than less formal wording. These results provide concrete guidance for researchers seeking to improve recruitment in real-world e-cohorts.

## Introduction

### Background and Rationale

Studies in the general population are essential to monitor the populations’ health status and to understand the etiology of medical conditions. Online cohorts (also known as e-cohorts) are a powerful tool to gather such data [1,2]. Indeed, compared to traditional forms of surveys such as in-person, postal, or telephone interviews, e-cohorts incur lower costs, reach larger sample sizes, limit geographic restrictions, and increase time efficiency with immediate submission and no manual re-entry, which is subject to errors [3,4]. However, e-cohorts have lower response rates than traditional ones [3,5-7], resulting in poorer representativity and lower reliability of results. Therefore, to ensure the future representativity of scientific research, it is essential to identify effective, low-cost strategies to increase participation rates in e-studies.

Such strategies include incentives, reminders, and alternative modes of data collection [8,9]. Among them, frequent electronic reminders can be cost-effective to increase completion rates (ie, inexpensive, fast, and can be automated), maintain engagement with participants, and enhance the quality of the data collected [8,10,11]. However, the effectiveness of these reminders may vary depending on several factors, such as the delivery mode (eg, text messages or emails) [10,12], the total number or time interval between reminders [13,14], or the content and formatting of messages, particularly emails (eg, formal institutional wording versus informal language) [6,15]. Furthermore, selecting an effective reminder strategy to increase representativity is challenging because participants differ widely in age and socioprofessional status. Those characteristics, associated with participation rates [3,7,16], might differently influence specific communication channels depending on device ownership, internet access, or digital literacy, for example. Relying on a single communication channel may not reach all subpopulations equally [2,4]; it is therefore necessary to tailor the strategy to participants’ preferences.

Data on the effectiveness of electronic reminders to increase participation rates in population-based e-cohorts of healthy adults are lacking. Indeed, most studies evaluating the benefits of electronic reminders are set in the context of in-person or postal questionnaires and might not directly apply to e-cohorts [9,11]. Most studies are nested within controlled trials or studies targeting specific populations (eg, specific medical conditions or university students) for which participants’ motivation or digital literacy, and thus participation rates, differ from studies in a nationwide population of healthy adults [3,6,12,17]. To our knowledge, no large-scale, randomized controlled trial has compared diverse delivery mode or format combinations (emails vs text messages and formal vs less formal wording) in a nationwide e-cohort of healthy adults. This lack of rigorously randomized evidence hampers the development of evidence-based guidelines for researchers seeking to improve recruitment in real-world e-cohorts.

### Objectives

Little is known about the best strategy for increasing participation and response rates in general population e-studies. Thus, the aim of this study was to evaluate which electronic reminder strategy was the most effective to optimize response rates in the context of a population-based e-cohort of healthy adults by conducting a randomized controlled trial nested in Le French Gut e-cohort [18]. We randomly assigned participants to receive 3 repeated reminders by email only (with either standard or institutional formatting), text message only, or a combination of the 3, and then measured response rates 30 days after the last reminder was sent. We hypothesized that a mixed delivery mode strategy combining emails and text messages, and a change of format from the first to the second email, would yield higher response rates than relying on a single channel [19,20].

## Methods

### Trial Setting, Eligibility Criteria, and Sample Size

We conducted a 4-arm randomized controlled trial nested in Le French Gut e-cohort. “Le French Gut – Le Microbiote Français” is an ongoing population-based, prospective, noninterventional e-cohort launched in September 2022 [18]. The aim of this national study is to describe the heterogeneity of the gut microbiota in the general population and to better understand its relationship with health, dietary habits, and lifestyle. The protocol for data collection has been previously described [18]. Briefly, participation is open to all metropolitan French adult residents and takes place exclusively online via a dedicated website [21], except for an at-home self-collected stool sample. To be enrolled in the cohort, volunteers must (1) register on the online platform (SKEZIA), (2) complete the eligibility questionnaire, and (3) sign the consent form, including consent to be contacted. To become active participants, enrolled individuals must then fill in the self-reported personal and lifestyle information questionnaire. Once the questionnaire has been completed, a stool sampling kit is automatically sent to the volunteer’s address.

In November 2024, we included all individuals who were enrolled in Le French Gut cohort (ie, those who registered online, providing their email address and phone number [optional]) but who were not yet active participants (ie, those who did not complete the eligibility questionnaire, consent form, and/or personal information questionnaire; the latter were included only if they had provided informed consent to be contacted). A total of 20,890 participants were eligible and included in this study. The trial was led by the SKEZIA team in charge of the online platform, without patient or public involvement. No other communication campaign promoted Le French Gut cohort during the time of the experiment.

### Ethical Considerations

The Le French Gut protocol was approved by the “Comité de Protection des Personnes” Sud-Est IV (21.00225.000006) and the “Commission Nationale Informatique et Liberté” (DR-2022‐141). Participants were included in the Le French Gut cohort only after they had provided their online informed consent. No compensation or incentives, such as gifts or personal feedback, were provided to participants. All data were deidentified to ensure confidentiality in compliance with French data protection regulations; as such, no identification of individual participants is possible in any part of the manuscript or its supplementary material. The study was conducted in accordance with the Helsinki principles and followed the 2025 CONSORT (Consolidated Standards of Reporting Trials) guidelines for reporting randomized trials (Checklist 1) [22].

### Trial Registration

The Le French Gut [23] study is registered in France under reference 2021-A01439-32 and on ClinicalTrials.gov (NCT05758961). The present nested trial was approved by the Le French Gut Scientific Committee and retrospectively registered on ClinicalTrials.gov (NCT07473466) on March 11, 2026. Prospective registration was not possible because the intervention was embedded in the operational workflow of the Le French Gut e-cohort after participants had already provided consent to be contacted. However, the study design, randomization algorithm, and outcomes were fixed before any reminders were sent, and the primary outcome (questionnaire completion) is an objective, automatically defined metric. The trial and analyses were led as prespecified in the protocol without modification. Consequently, the risk of bias being introduced by delayed registration is minimal. Furthermore, prospective registration was not required according to the ICMJE (International Committee of Medical Journal Editors) guidelines because no health-related outcomes were measured [24].

### Trial Design

**Figure 1.**
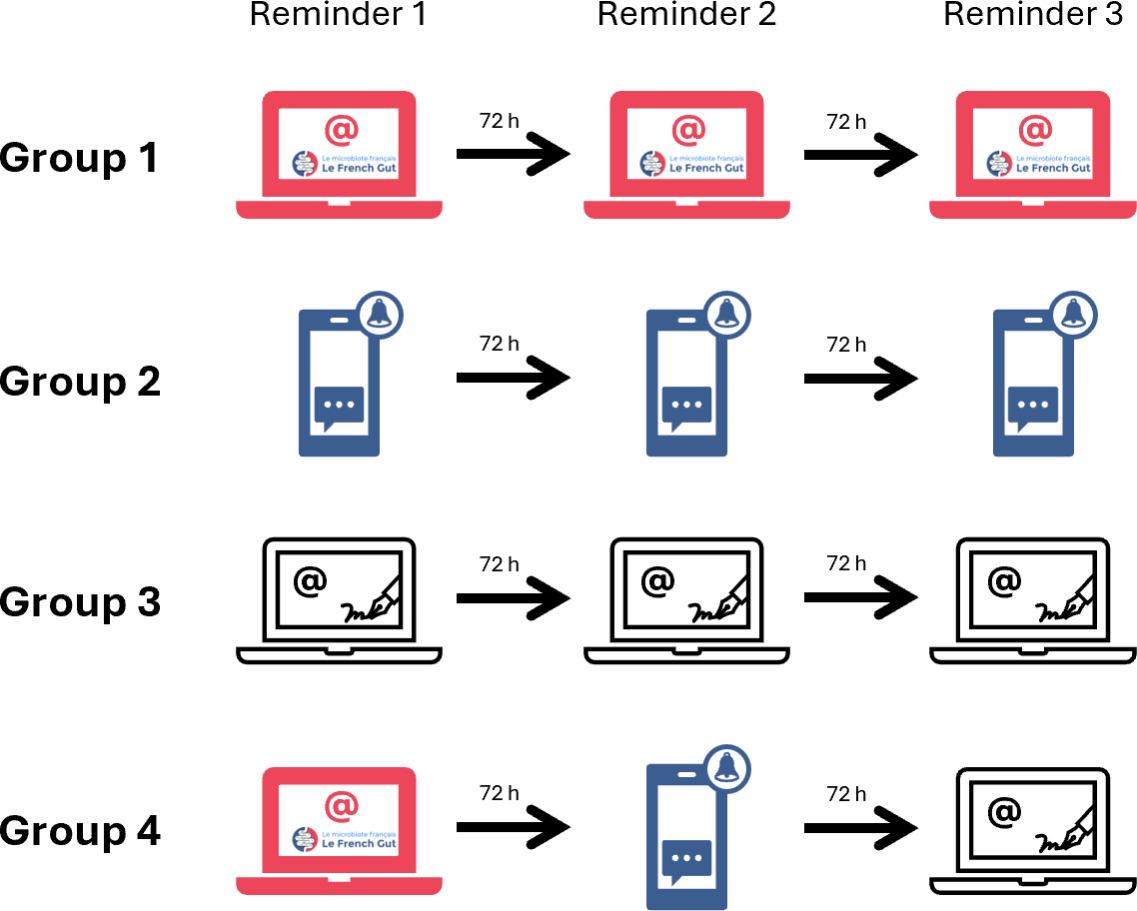
Trial design with 4 experimental groups with different delivery modes and/or reminder formats, randomized controlled trial nested in Le French Gut, France, 2024. Participants were randomly allocated to 1 of 4 groups, which differed in terms of delivery mode and/or formatting of the reminders. Three reminders were sent, each spaced 72 hours apart. In group 1, participants received 3 identical standard emails. In group 2, they received 3 identical text messages. In group 3, participants received 3 identical “institutional” emails, with formal wording and signed by the study coordinators. In group 4, we used a mixed delivery approach, combining a standard email, followed by a text message, and finally an “institutional” email. Once participants had completed the questionnaire, they were removed from the contact list and did not receive the following reminders.

The experiment was conducted exclusively online and involved sending 3 reminders, each spaced 72 hours apart, inviting participants to complete the online personal information questionnaire to validate their participation in Le French Gut. Participants were randomly allocated to 1 of 4 parallel groups, which differed in terms of delivery mode and/or formatting of the reminders: (group 1) standard (nonformal) emails only; (group 2) text messages only; (group 3) “institutional” emails only (ie, formal wording and signed by the study coordinators); (group 4) a mixed delivery approach, combining a standard email, followed by a text message, and finally an “institutional” email (Figure 1). The reminders were not personalized; they all contained the same link to the online platform and were sent on the same day to all participants. The reminders were written and formatted by the Le French Gut researchers in charge of the trial and then programmed by the technical team (ie, SKEZIA developers) using Tipimail (Positive Group, France) for emails and smsmode (Calade Technologies) for text messages. Once participants had fully completed the questionnaire, they were removed from the mailing list for subsequent reminders. The wording used in all 3 types of reminders is presented in Multimedia Appendix 1.

### Randomization

The eligible participants were randomly assigned to 1 of 4 experimental groups using a centralized computer-generated randomization. The randomization was performed on Python by a statistician. To ensure comparability between groups, randomization was stratified by time since online registration (<6, 6‐12, 12‐18, 18‐24, and >24 mo). Briefly, within each stratum, participants were randomly permuted, using a fixed seed to ensure reproducibility; the permuted list was then evenly divided into 4 groups (ratio 1:1:1:1), and 1 of the 4 interventions was assigned to each group. The randomization file was sent to the technical team in charge of sending the reminders via the email sender software Tipimail (Positive Group) or text message sender software smsmode (Calade Technologies) according to the allocation groups. The participants were unaware that the trial was being conducted, and the researchers and data analysts were blind to participants’ assigned groups.

### Data Collection

At inclusion, gender and age were collected from the personal information questionnaire when available (ie, a subset of participants had started but not finished this questionnaire at the start of this study; therefore, some data were missing). For each participant, data collected from the SKEZIA platform included: the status regarding completion of their personal information questionnaire 30 days after the last reminder (not started, in progress, or fully completed); the date of questionnaire completion, if fully completed; and the date of first login on the platform after the last sent reminder, if available. We also retrieved the numbers of emails delivered, opened, and clicked from the email sender software Tipimail (Positive Group, France). Due to technical limitations, similar metrics could not be retrieved for text messages. No harm to participants or unintended events were defined or assessed (systematically or nonsystematically) as the intervention (sending reminders for completion of a questionnaire) was not a health-related measure and had no direct impact on the participants.

### Outcomes

The primary outcome was the completion rate of the personal information questionnaire 30 days after the last reminder was sent, calculated as follows: number of participants who fully completed the questionnaire divided by the total number of participants included in the study.

Secondary outcomes included (1) completion time: time (in hours) between the date the last reminder was sent to a participant and the date of questionnaire completion, (2) login rate: number of participants who logged into their account divided by the total number of participants included in the study, (3) login time: time (in hours) between the date the final reminder was sent to a participant and the date of their next login to the platform, (4) email opening rate: number of opened emails divided by the number of delivered emails, (5) email click-through rate: number of participants who clicked on the link in the email divided by the number of opened emails. No missing data were observed for the outcomes in our study.

### Statistical Analysis

We checked the adequacy of the stratified randomization process by describing the distribution of time since online registration across the 4 groups. We also evaluated randomization’s robustness by comparing gender and age between groups when information was available.

We tested which reminders’ delivery mode and/or formatting optimized response rate by comparing the questionnaire completion rates across the 4 groups. We compared the overall completion rates between the first, second, and third reminders. We compared the groups in terms of completion time, login rate, and login time. Finally, we evaluated the differences in email opening and click-through rates between standard and institutional emails. Chi-square tests were performed to compare categorical variables between groups, and ANOVAs were used for continuous variables. A significant level of .05 was applied for all statistical tests. Statistical analyses were performed using RStudio (version 4.3.1; R Foundation for Statistical Computing).

Due to the lack of contact information, some participants could not be reached through the communication mode of the group they were allocated to; thus, both per-protocol (PP) and intention-to-treat analyses are presented.

## Results

### Participant Flow and Randomization

**Figure 2.**
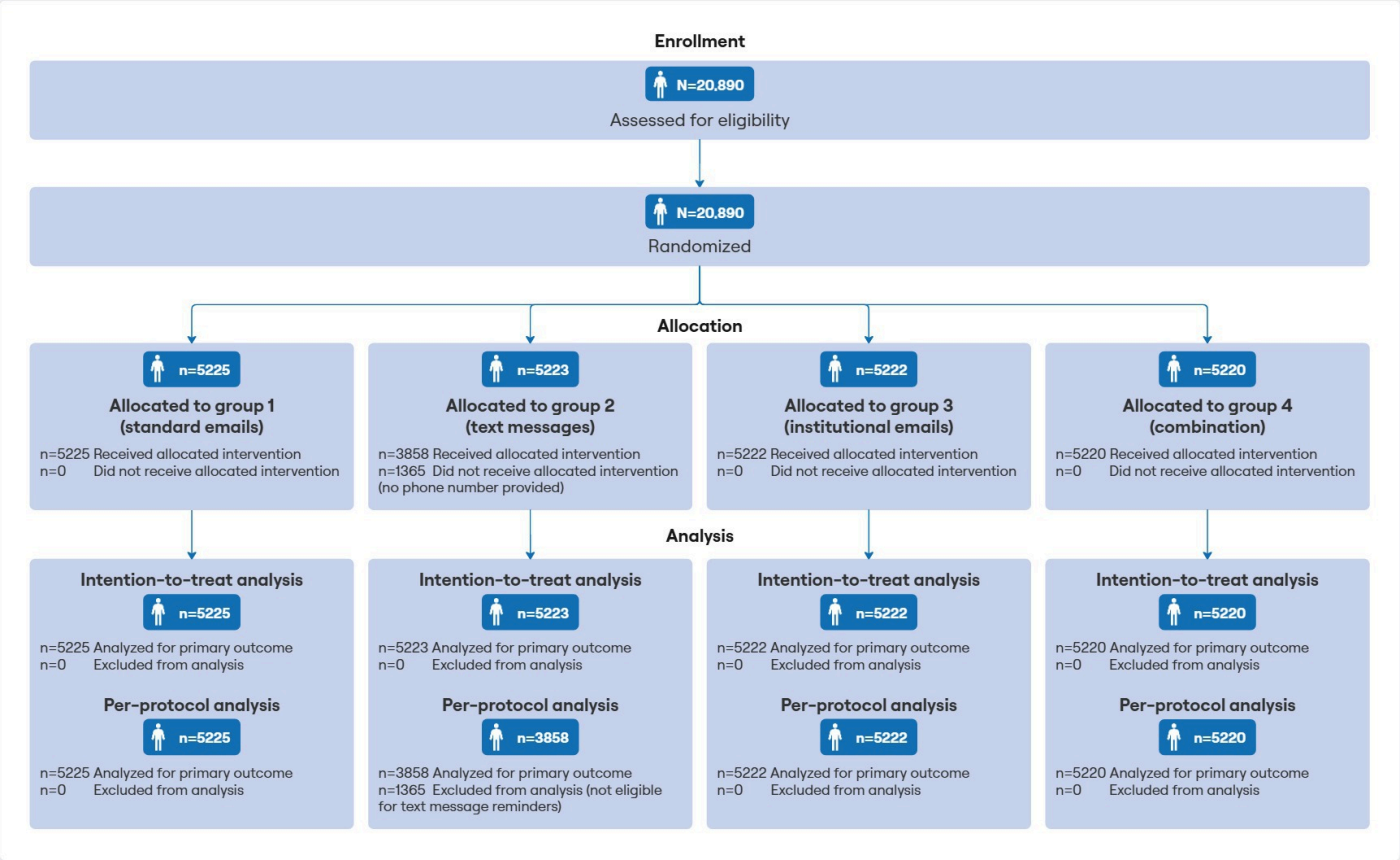
Flowchart of the participants included in the study for intention-to-treat (N=20,890) and per-protocol (N=19,525) analysis, randomized controlled trial nested in Le French Gut, France, 2024.

In November 2024, a total of 20,890 volunteers from Le French Gut were included in this study (Figure 2) and were randomly assigned to 1 of the 4 groups as follows: 5225 participants in group 1; 5223 in group 2; 5222 in group 3; and 5220 in group 4. Among them, 33% (n=6889) had been registered in Le French Gut for more than 2 years before the start of this study, 5.3% (n=1103) for 18 to 24 months, 22.4% (n=4687) for 12 to 18 months, 26.5% (n=5533) for 6 to 12 months, and 12.8% (n=2678) for less than 6 months (Table 1).

**Table 1.** Baseline participants’ characteristics by experimental group, randomized controlled trial nested in Le French Gut, France, 2024 (N=20,890).

Characteristic	Total (N=20,890)	Group 1 (standard emails; n=5225)	Group 2 (text messages; n=5223)	Group 3 (institutional emails; n=5222)	Group 4 (combination; n=5220)	*P* value^a^
Stratification variable						
Time since registration (mo), n (%)						.99
<6	2678 (12.8)	670 (12.8)	670 (12.8)	669 (12.8)	669 (12.8)	
6‐12	5533 (26.5)	1384 (26.5)	1383 (26.5)	1383 (26.5)	1383 (26.5)	
12‐18	4687 (22.4)	1172 (22.4)	1172 (22.4)	1172 (22.4)	1171 (22.4)	
18‐24	1103 (5.3)	276 (5.3)	276 (5.3)	276 (5.3)	275 (5.3)	
>24	6889 (33.0)	1723 (33.0)	1722 (33.0)	1722 (33.0)	1722 (33.0)	
Demographic characteristics^b^						
Participants, n	5984	1461	1394	1564	1565	
Age (years), mean (SD)	47.6 (14.3)	47.0 (14.4)	48.0 (14.3)	47.9 (14.2)	47.5 (14.4)	.37
Women, n (%)	3599 (78.0)	896 (77.4)	870 (80.6)	912 (76.8)	921 (77.3)	.13

a *P* values from chi-square tests for time since registration and sex, and ANOVA for age.

b Demographic characteristics were available for a subset of participants who had started, but not finished, the personal information questionnaire at the start of this study (n=5984). Within this subset, means and percentages of nonmissing values are presented. Missing values: 22.3% for age (20.5%, 22.0%, 23.5%, and 23.2% in groups 1 to 4, respectively), and 22.8% for sex (20.7%, 22.5%, 24.0%, and 23.9% in groups 1 to 4, respectively).

Data on age and gender were available, at the time of randomization, for a subset of 5984 participants, with balanced numbers of participants across the 4 groups (Table 1). The mean age was 47.6 (SD 14.3) years, and 78.0% (n=3599) of the participants were women. These characteristics were similar across the 4 groups (*P*=.37 for age and *P*=.13 for gender). The balanced group sizes and similar distributions of time since registration (ie, stratification variable), age, and gender across groups reflect the robustness of the random allocation procedure.

Among the 20,980 participants, 5434 had not provided a phone number, with similar proportions across groups (25.0% [n=1307] of the participants in group 1, 26.1% [n=1365] in group 2, 26.5% [n=1382] in group 3, and 26.4% [n=1380] in group 4; *P*=.29). Considering that a phone number was required to receive text message reminders in group 2, 1365 participants from group 2 were not considered in the PP analysis (Figure 2). Thus, the PP analysis included 19,525 participants.

### Primary Outcome: Questionnaire Completion

By the end of the study, 30 days after the last reminder was sent, 1634 participants had fully completed the personal information questionnaire, corresponding to 8.4% of the 19,525 participants in the PP analysis (Table 2). Completion rates differed between groups (*P*=.005), with the highest rate observed in group 4 (490/5220, 9.4%), then groups 2 (323/3858, 8.4%) and 3 (432/5222, 8.3%), and the lowest completion rate in group 1 (389/5225, 7.4%). The intention-to-treat analysis yielded similar results (*P*<.001), that is, the highest completion rate was in group 4 (490/5220, 9.4%), but the lowest was in group 2 (323/5223, 6.2%). Completion rates did not differ between the first, second, and third reminders, each yielding completion rates of about 3% (PP analysis: 601/19,525, 3.1%, 491/17,573, 2.8%, and 542/18,433, 2.9%, respectively; *P*=.27). Among participants who fully completed the personal information questionnaire, age and gender distributions did not differ across the 4 groups (*P*=.56 for age and *P*=.13 for gender; Table 3). However, time since registration differed (*P*<.001). The proportion of respondents who had been registered for more than 1.5 years was higher in group 2 compared with the other groups, while group 1 had more participants who had been registered for less than 6 months.

**Table 2.** Completion rates of the personal information questionnaire by experimental group, randomized controlled trial nested in Le French Gut, France, 2024 (N=20,890).

Outcome	Total	Group 1 (standard emails)	Group 2 (text messages)	Group 3 (institutional emails)	Group 4 (combination)	Chi-square test *P* value
Per-protocol analysis						
Analysis population, n	19,525	5225	3858	5222	5220	—^a^
Fully completed questionnaire, n (%)	1634 (8.4)	389 (7.4)	323 (8.4)	432 (8.3)	490 (9.4)	.005
Intention-to-treat analysis						
Analysis population, n	20,980	5225	5223	5222	5220	—
Fully completed questionnaire, n (%)	1634 (7.8)	389 (7.4)	323 (6.2)	432 (8.3)	490 (9.4)	<.001

a Not applicable.

**Table 3.** Characteristics of participants who completed the personal information questionnaire by experimental group, randomized controlled trial nested in Le French Gut, France, 2024 (N=20,890).

Characteristic	Total (N=1634)	Group 1 (standard emails; n=389)	Group 2 (text messages; n=323)	Group 3 (institutional emails; n=432)	Group 4 (combination; n=490)	*P* value^a^
Time since registration (months), n (%)						<.001
<6	164 (10.0)	54 (13.9)	12 (3.7)	45 (10.4)	53 (10.8)	
6‐12	405 (24.8)	104 (26.7)	63 (19.5)	121 (28.0)	117 (23.9)	
12‐18	441 (27.0)	99 (25.4)	92 (28.5)	109 (25.2)	141 (28.8)	
18‐24	108 (6.6)	24 (6.2)	30 (9.3)	25 (5.8)	29 (5.9)	
>24	516 (31.6)	108 (27.8)	126 (39.0)	132 (30.6)	150 (30.6)	
Age (years), mean (SD)	49.4 (13.6)	49.8 (13.3)	49.8 (13.8)	49.5 (13.2)	48.7 (14.0)	.56
Women, n (%)	1192 (72.9)	292 (75.1)	248 (76.8)	305 (70.6)	347 (70.8)	.13

a *P* values from chi-square tests for time since registration and sex, and ANOVA for age.

### Secondary Outcomes

#### Completion Time

The mean completion time for the personal information questionnaire was 6.9 (SD 3.9; range 0‐18) days. The completion times differed between groups (*P*=.003), with a faster completion in group 2 (mean 6.3, SD 4.3 d) compared with groups 3 and 4 (mean 7.0, SD 3.5 d and mean 7.2, SD 3.8 d, respectively); and it was 6.6 (SD 3.9) days in group 1.

#### Login Rate and Time

A total of 2795 participants logged into their accounts between the day of the first reminder and the end of the study, representing 14.3% of the study population who received a reminder (PP analysis). Login rates differed across the 4 groups (*P*<.001): 12.1% (632/5225) of participants in group 1, 14.3% (553/3858) in group 2, 15.3% (798/5222) in group 3, and 15.6% (812/5220) in group 4. The mean login time was 6.7 (SD 4.2; range 0‐18) days, and it differed between groups (mean 6.7, SD 4.3, mean 6.2, SD 4.6, mean 6.8, SD 3.9, and mean 7.1, SD 4.3, for groups 1 to 4, respectively; *P*=.002).

#### Email Opening and Click-Through Rates

During the study period, a total of 20,487 standard emails were sent. Among these emails, 8459 were opened, representing an opening rate of 41.3%, of which 1654 were clicked, corresponding to a click-through rate of 19.6% for opened emails (8.1% of total sent emails). Similarly, among the 20,109 institutional emails sent, 8286 were opened, representing an opening rate of 41.2%, and 1917 of those were clicked, corresponding to a click-through rate of 23.1% for opened emails (9.5% of sent emails). The opening rate did not differ between the standard and institutional emails (*P*=.87), but the click-through rate for institutional emails was higher than for standard emails (*P*<.001).

#### Deleted Accounts

Twenty-one of 20,890 (0.10%) participants deleted their account during the study; the rate did not differ between groups (*P*=.27).

## Discussion

### Principal Findings

We conducted a 4-arm randomized controlled trial to evaluate the effectiveness of different reminder strategies in increasing response rates to an e-study. We compared 4 groups differing only in terms of delivery mode (ie, emails or text messages) and/or formatting of the emails (ie, standard or institutional wording). Our results clearly show that emails and text messages can be used to improve e-cohort participation and give indications of the advantages of different reminder strategies. First, as hypothesized, we found that a mixed delivery mode strategy, combining emails and text messages as well as standard and institutional email wording, yielded the highest completion rate. This higher response rate was in line with the higher login rate observed in this group, indicating that using diverse channels is effective in reaching participants. Second, text messages prompted faster responses compared with emails, and they seemed to be more effective in reaching participants who had been inactive for a long time (ie, registered for more than 1.5 y), suggesting that they are optimal for re-engaging long-term inactive e-cohort participants. Third, the formatting of emails did not influence their opening rates, but institutional emails, with formal wording and signed by study coordinators, had a higher click-through rate than standard, less formal ones.

### Comparison With Prior Work

Our results are in line with the few studies that investigated reminder strategies in the context of e-surveys [3], indicating that electronic reminders, especially emails, are effective in increasing response rates [25]. However, the optimal strategy may vary depending on population characteristics, technological access, or message wording or design [6]. Previous studies have shown that multiple reminders enhance response rates in e-surveys, though their incremental impact may diminish with each subsequent contact [25-27]. For example, in a Belgian e-cohort of 15,651 university students, the first reminder email increased the response rate by 10.3%, while the second and third reminders contributed to smaller gains (8.6% and 6.1%, respectively) [26]. In contrast, our nationwide general population study found that each of the 3 reminders yielded a consistent ~3% increase in completion rates. This difference may reflect variations in participant engagement: university students, who are often more digitally literate and connected [16], may respond more strongly to initial prompts, whereas the general population may require sustained encouragement. Studies also show that email formatting is important, with higher response rates achieved through personalization (eg, addressing the recipient by name) [25,27,28] and change of wording between reminders [25]. It is worth noting that these studies primarily involved younger, highly educated populations (eg, university students in Spain [27,28], Belgium [26], or the United States [25]) who may differ from the broader demographic in our cohort [25]. Our results suggest that formal institutional emails (signed by study coordinators) had higher click-through rates than standard emails, indicating that perceived authority or credibility may be important in a general adult population.

Few studies have focused on text message reminders, highlighting their efficacy in improving response rates, which may vary depending on the study setting [29-35]. A study of 16,739 working-age adults in Finland found that text message reminders yielded higher response rates than postal reminders [32]. Similar to previous studies, our data show that text messages prompted faster responses, which may reflect a distinctive psychological impact of text messages, which are convenient and accessible: they are brief, appear as personal “push” notifications, are perceived as more immediate than emails, and are not subject to spam filters [29-31]. Thus, text messages can be especially useful when rapid response is required [34]. However, population-level factors, such as demographics and access characteristics, may influence the effectiveness of text message reminders [34,36]. For example, among 3876 Swedish employees, high internet availability amplified the effectiveness of frequent reminders, and office workers responded more to reminders than field workers in an e-survey on lifestyle habits [33].

While prior work has examined reminder strategies in specific populations (eg, students, clinical cohorts), our randomized controlled trial is among the first to rigorously compare multiple reminder strategies (delivery mode and wording) in a large nationwide population-based e-cohort of healthy adults. By embedding our trial within an ongoing cohort, we provide real-world evidence on how reminder strategies perform in a general population setting, where digital literacy, motivation, and access vary widely. Our findings underscore the importance of tailoring reminder strategies to population characteristics and highlight the value of mixed delivery approaches for maximizing participation, a major challenge in observational research [3].

### Limitations

The higher response rate in the combination group was not driven by differences in age or gender. However, due to the lack of information, we were not able to assess other socioeconomic markers, such as education or health conditions, which might influence selective participation [7,16]. Moreover, our analysis did not consider the stage of the inclusion process that the participant had reached before the start of the experiment (ie, eligibility, consent, or personal information questionnaire). Depending on the initial stage, completing the questionnaire requires varying numbers of steps, which could influence completion rates. However, Le French Gut questionnaires were meticulously designed to ensure completion in under 10 minutes, thereby decreasing the burden on participants. Randomization also mitigates this potential bias.

Furthermore, there are several practical limitations specific to the use of text messages. First, investigators should weigh the potential costs associated with sending text message reminders against the modest increase in response rate. In our study, each text message had an approximate raw cost of ~€0.05 (excluding platform fees; €1=US $1.15 as of July 31, 2026), and the mixed delivery mode strategy (including 1 text message) yielded an absolute gain of 1% to 2% compared with email-only strategies. In a sample of 10,000 potential participants, this would represent roughly 100 to 200 additional responses at an incremental cost of about €500 (ie, ~€2.5 to €5 per extra respondent). Second, relying only on text messages is likely to induce a selection bias. Indeed, 25.9% (5434/20,980) of the participants did not provide a phone number and were thus excluded from the PP analysis. Thus, a reminder strategy based only on text messages is likely to exclude participants, either because they do not own a mobile phone or because they chose not to share their number, potentially introducing selection bias. Finally, due to technical limitations, we had no data on text message opening and click rates, limiting the comprehensive understanding of this mode of delivery.

Contrary to text messages, emails are virtually free and can be delivered to all participants. However, emails have their own limitations, such as reduced visibility in overloaded inboxes and a risk of being spam-blocked [37]. Overall, the advantage of the mixed delivery mode strategy in increasing response rate likely arises from the complementary strengths of each channel, where the limitations of one are counterbalanced by the strengths of the other. This balancing effect may also explain why the mixed delivery mode achieved the highest completion rate despite the slower completion time. This discrepancy might be due to the sequential ordering of the reminders: first, the standard email (similar to group 1’s moderate response time); second, the text message (similar to group 2’s fastest response); and third, the institutional email (similar to group 3’s slower response). A different reminder sequence could have yielded a faster response while maintaining high completion rates, warranting further investigation. Despite the use of 3 reminders, spaced 72 hours apart, the overall completion rate was only 8.4% (1634/19,525), corresponding to 58.5% (1634/2795) of participants who logged into their account during the experiment. This overall modest response rate might be explained by (1) the fully online format of the study, which has the advantage of being cheaper and easier to implement but might be subject to specific barriers such as survey fatigue or messages being spam-blocked [38]; (2) the target population, as the general healthy population might not be as involved in the research as would be a clinical population targeted for a self-related specific health concern with potential personal benefits of the research [39,40]; (3) the time since registration, with most participants having first registered online more than 2 years before the experiment and thus might not remember the context and objectives of the Le French Gut cohort or even that they previously registered for it.

### Implications

Our study focused on increasing the participation rate during the inclusion process of an observational study. To be applicable to attrition and retention rates in longitudinal e-cohorts, a mixed delivery mode strategy should be tested in other contexts, such as recontacting participants for second-round or follow-up questionnaires. Such a study in a longitudinal setting will also clarify whether reminders alone can increase response rates among participants who are already involved in the study or whether additional approaches (such as in-person reminders, phone calls, financial or other incentives, gamification, etc) are required to maintain long-term participation in e-cohorts. These combined strategies should be further investigated. Future work could also explore how patient-oriented software or apps could incorporate reminder systems to optimize patient engagement.

### Conclusions

Altogether, our large-scale, specifically designed, randomized controlled trial showed that electronic reminders can be a useful tool to increase the response rate in the context of a population-based e-cohort of healthy adults. Specifically, a mixed delivery mode strategy, combining emails and text messages, reached more participants than single-channel strategies; text messages prompted faster responses and were effective in reaching long-term inactive participants; and formally worded emails signed by the study coordinator seemed more appealing to participants than less formal wording. Therefore, when planning the reminders, we strongly advise researchers to carefully consider the contact channel and formatting of messages while also assessing the cost-effectiveness of each strategy and the limitations of relying on a single strategy. While previous studies have often focused on specific populations like students or clinical cohorts, our trial uniquely included a general adult population and directly compared multiple reminder strategies in the same population, contributing to new knowledge and providing concrete, actionable guidance for researchers seeking to improve recruitment in real-world e-cohorts.

## Supplementary material

10.2196/88577 Multimedia Appendix 1 Wording used in the reminders for standard emails, institutional emails, and text messages, randomized controlled trial nested in the Le French Gut, France, 2024.

10.2196/88577 Checklist 1 CONSORT checklist.

## Abbreviations

CONSORT: Consolidated Standards of Reporting Trials
ICMJE: International Committee of Medical Journal Editors
PP: per-protocol
