# Supplementary material for: Mixed Delivery Mode Reminder Strategy to Tackle Low Participation in an e-Cohort: Randomized Controlled Trial Nested Within the Le French Gut e-Cohort
Source: J Med Internet Res. 2026 Aug 18;28:e88577. doi: 10.2196/88577 (PMC13483751; doi:10.2196/88577)
Supplement: Supplementary file 1 [file jmir-v28-e88577-s001.pdf]

Supplementary Table: Wording used in the reminders for standard emails, institutional emails and text messages, randomized-controlled trial nested in Le French Gut, France, 2024

Type of reminder	English translation	Original French version
**Standard email**	Subject: Le French Gut - It's still time to participate!<br><br>Dear participant,<br><br>Thank you for registering for the Le French Gut project! Your commitment is essential to advance research on intestinal microbiota.<br><br>We noticed that you have not yet completed the questionnaire or provided your stool sample. These steps are crucial for your participation to be complete and for us to make the most of this study.<br><br>By actively taking part, you help us better understand the role of microbiota in health and lay the groundwork for future medical solutions to better prevent and treat chronic diseases. We count on you!<br><br>To complete your journey, please visit www.lefrenchgut.fr now in your participant portal. This will take only a few minutes.<br><br>Thank you again for your valuable contribution to this great scientific adventure!<br><br>Best regards,<br><br>The Le French Gut Team	Objet : Le French Gut - Il est encore temps de participer !<br><br>Chère participante, cher participant,<br><br>Merci de vous être inscrit au projet Le French Gut ! Votre engagement est essentiel pour faire avancer la recherche sur le microbiote intestinal.<br><br>Nous avons remarqué que vous n'avez pas encore complété le questionnaire ni effectué votre don de selles. Ces étapes sont cruciales pour que votre participation soit complète et pour que nous puissions tirer le meilleur parti de cette étude.<br><br>En participant activement, vous contribuez à mieux comprendre le rôle du microbiote sur notre santé et au futur développement de solutions médicales pour mieux prévenir et soigner les maladies chroniques. Nous comptons sur vous !<br><br>Pour terminer votre parcours, rendez-vous dès maintenant sur www.lefrenchgut.fr dans votre espace participant. Cela ne prendra que quelques minutes.<br><br>Merci encore pour votre précieuse contribution à cette grande aventure scientifique !<br><br>Cordialement,<br><br>L'équipe Le French Gut

<table>
<tr>
<td>Institutional email</td>
<td><u>Subject: Le French Gut - Your participation is essential for tomorrow's health</u>

Dear participant,

I would like to sincerely thank you for your commitment to the Le French Gut project - the French microbiota. Your contribution plays a fundamental role in this study that will help us better understand the impact of intestinal microbiota on our health.

Today, it is crucial to complete your journey by filling out the questionnaire and providing your stool sample. These data are essential to accelerate research in order to better prevent and treat chronic diseases.

Your support helps build a unique knowledge that could revolutionize tomorrow's medicine and the development of innovative treatments. We count on you to complete your participant journey.

We count on you to finalize your contribution and join the numerous other participants. To complete your journey, please visit www.lefrenchgut.fr now in your participant portal. This will take only a few minutes.

Thank you again for your valuable participation and your commitment to scientific research.

Sincerely,

Prof. XXX

Head of Gastroenterology and Digestive Oncology Service at Hospital XXX

Investigator physician of the Le French Gut project</td>
<td><u>Objet : Le French Gut - Votre participation est essentielle pour la santé de demain</u>

Chère participante, cher participant,

Je tiens à vous remercier sincèrement pour votre engagement dans le projet Le French Gut – le microbiote français. Votre contribution joue un rôle fondamental dans cette étude qui permettra de mieux comprendre l'impact du microbiote intestinal sur notre santé.

Aujourd'hui, il est crucial de compléter votre parcours en remplissant le questionnaire et en réalisant votre don de selles. Ces données sont essentielles pour accélérer la recherche afin de mieux prévenir et soigner les maladies chroniques.

Votre soutien permet de construire une base de connaissances unique qui pourrait révolutionner la médecine de demain et le développement de traitements innovants. Nous comptons sur vous pour terminer votre parcours participant.

Nous comptons sur vous pour finaliser votre contribution et rejoindre les nombreux autres participants. Pour terminer votre parcours, rendez-vous dès maintenant sur www.lefrenchgut.fr dans votre espace participant. Cela ne prendra que quelques minutes.

Merci encore pour votre précieuse participation et votre engagement envers la recherche scientifique.

Cordialement,

Pr. XXX

Chef du service de gastro-entérologie et oncologie digestive de l'hôpital XXX.

Médecin investigateur du projet Le French Gut</td>
</tr>
</table>

**Text message**	We need you!<br>Your participation in the Le French Gut project is essential to revolutionize microbiome research. Continue your journey: complete the questionnaire and submit your stool donation right now at www.lefrenchgut.fr in your participant portal. We're counting on you, together we can change everything!	On a besoin de vous !<br>Votre participation au projet Le French Gut est essentielle pour révolutionner la recherche sur le microbiote ! Continuez votre parcours : remplissez le questionnaire et faites votre don de selles dès maintenant sur www.lefrenchgut.fr dans votre espace participant. On compte sur vous, ensemble on peut tout changer !
